# Mandibular First Molar with a Single Root and Single Canal

**DOI:** 10.1155/2014/159846

**Published:** 2014-02-20

**Authors:** Chandrasekaran Sooriaprakas, Suma Ballal, Natanasabapathy Velmurugan

**Affiliations:** Department of Conservative Dentistry and Endodontics, Meenakshi Ammal Dental College and Hospital, Meenakshi Academy of Higher Education and Research (MAHER), Alapakkam Main Road, Maduravoyal, Chennai, Tamil Nadu 600 095, India

## Abstract

Successful endodontic management of mandibular first molar with a single root and single canal is diagnosed with the aid of dental operating microscope and multiple angled radiographs. In addition all the mandibular molars and premolars were single rooted on either side.

## 1. Introduction

A thorough knowledge of root canal anatomy is necessary to achieve appropriate cleaning and shaping of the root canal system and ensure success of endodontic treatment [1]. Very often, the mandibular first molars require endodontic treatment as they are the first permanent posterior teeth to erupt and are commonly affected by caries [2]. Typically, the mandibular first molar presents with 2 well-defined roots: a mesial root with two canals and a distal root with one or two canals. Variations in the form, configuration, and number of root canals in mandibular molars have been discussed extensively in endodontic literature [3, 4]. These include mandibular first molar with five, six, and seven root canals [5–7], middle mesial canal [8], middle distal canal [9], four canals in mesial root [10], four canals in distal root [11], radix entomolaris [12], and “C-” shaped canal [13]. These reports predominantly include cases with more number of canals than normal.

However, the clinician should also be aware of the possibility of the existence of lesser number of roots or canals. Gopikrishna et al. published a case of single root with a single canal in a maxillary first molar [14]. Recently, Krithikadatta et al. have reported a case of a mandibular first molar with two roots and two root canals [15]. The purpose of this paper is to report the uncommon anatomy of a mandibular first molar with a single root and single canal, which has not been reported in endodontic literature.

## 2. Case Report

**Figure 1:**
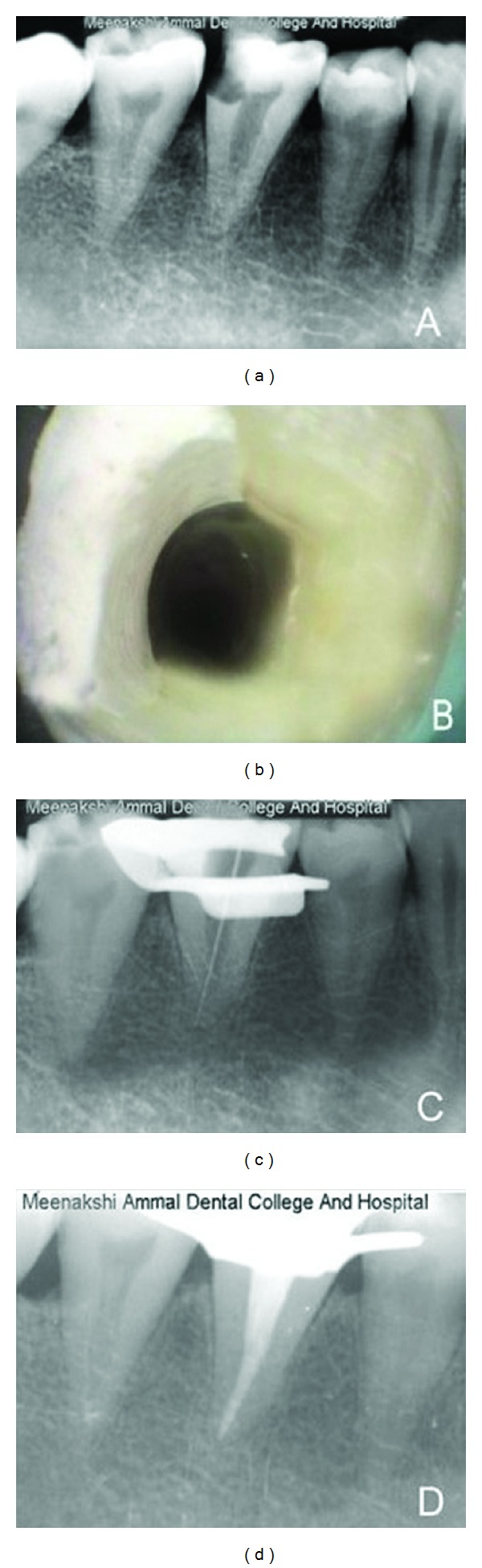
(a) Preoperative radiograph showing the posterior teeth with single root. (b) Access opening demonstrating single canal. (c) Working length radiograph. (d) Postobturation radiograph.

A 28-year-old male patient with the chief complaint of spontaneous pain in the lower right posterior tooth was referred for root canal treatment. History revealed intermittent pain for the past 1 month, which had increased in intensity for the past 4 days. Subjective symptoms included prolonged sensitivity to thermal stimuli and an increase in intensity of pain, which awakened the patient throughout the night. The patient's medical history was noncontributory. Clinical examination of the right mandibular first molar revealed the presence of a large distoocclusal carious lesion which was tender on percussion and also the presence of a single root. Periodontal probing around the tooth and mobility were within physiological limits. Vitality testing with dry ice (RC Ice; Prime Dental Products Pvt. Ltd., Mumbai, India) caused an intense lingering pain, whereas electric pulp testing (Parkell Electronics Division, Farmingdale, NY) showed exaggerated response. Preoperative radiographs revealed a distoocclusal radiolucency approaching the pulp space with a widened periodontal ligament space adjacent to the root apex. Multiple angulated radiographs also confirmed the presence of a single root and a single canal (Figure 1(a)). From the clinical and radiographic examination, a diagnosis of symptomatic irreversible pulpitis with symptomatic apical periodontitis was made and hence routine nonsurgical endodontic treatment was planned.

Local anesthesia was induced using 1.8 mL 2% lidocaine with 1 : 200,000 epinephrine (Xylocaine; AstraZeneca Pharma India Ltd., Bangalore, India). Following caries excavation the distal surface of the tooth was restored with IRM (IRM; Dentsply De Trey GmbH, Konstanz, Germany). Rubber dam was placed and a conventional endodontic access opening was established with an Endo Access bur (Dentsply Tulsa, Tulsa, OK). On access opening, a single large canal was located in the centre of the pulp chamber. Usually a single rooted mandibular molar could be associated with C-shaped canal but in this case a “C-” shaped orifice/canal was not identified, instead a single large canal was present at the centre of the pulp chamber which was confirmed using dental operating microscope (Seiler Revelation, St. Louis, MO, USA) (Figure 1(b)).

Working length was determined using radiographs (Ingle's method) and confirmed with an apex locator (Root ZX II, Morita, Tokyo, Japan) (Figure 1(c)). Cleaning and shaping was done using circumferential filing technique with ISO 2% taper files up to size 60 (MANI Inc., Tochigi-Ken, Japan). Irrigation was performed using normal saline (Nirma Pvt. limited, Gujarat, India), 2.5% sodium hypochlorite solution, and 17% EDTA (Prime Dental Product Pvt. Ltd., Mumbai, India). Final rinsing of the canal was performed using 2% chlorhexidine digluconate coupled with ultrasonic agitation. The canal was dried with absorbent points (Dentsply Maillefer Instruments, Ballaigues, Switzerland) and obturation was performed using cold lateral compaction of gutta-percha (Dentsply Maillefer Instruments, Ballaigues, Switzerland) and AH Plus resin sealer (Maillefer Dentsply, Konstanz, Germany) (Figure 1(d)). After completion of root canal treatment, the tooth was restored using resin composite (Z250; 3M ESPE Dental Products, St. Paul, MN). The patient was asymptomatic during the followup period of one year.

## 3. Discussion

**Figure 2:**
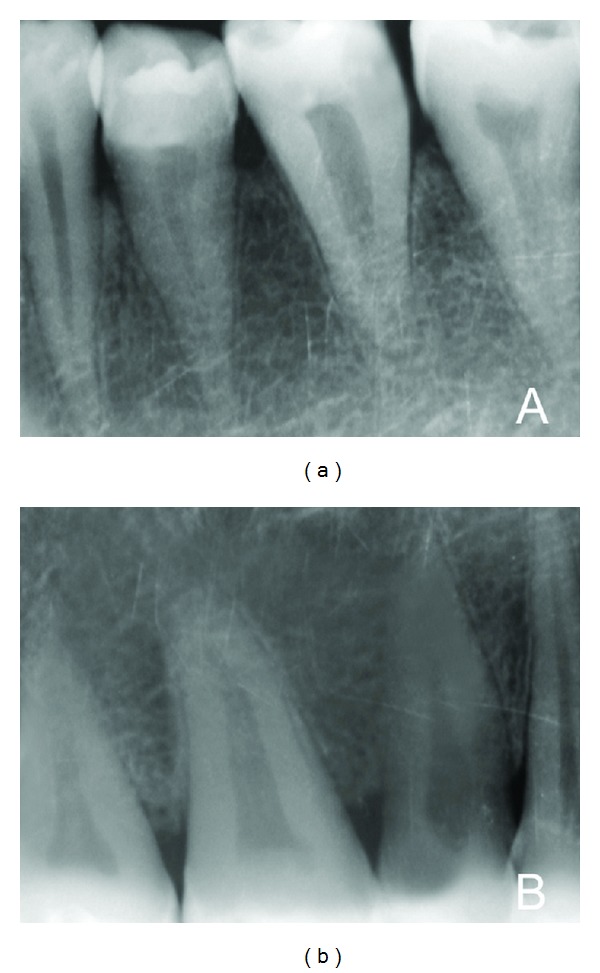
(a) Radiograph shows all the mandibular posterior teeth: contralateral side. (b) Radiograph shows all the right maxillary posterior teeth with single root.

Our case report highlights the presence of an unusual anatomy in mandibular first molar that had a single root and a single canal. Sabala et al. [16] stated that the rarer the aberration is, the greater the probability of it being bilateral will be. Interestingly in the present case, all the mandibular posterior teeth, both premolars and molars, had single root canal and single canal (Figure 2(a)). Also the right upper molars and premolars had single root and single canal (Figure 2(b)). Left maxillary posteriors were extracted and hence the morphology could not be determined. Fava et al. [17] had identified the existence of such anatomical variation in second molars in all the maxillary and mandibular second molars. Majority of the permanent mandibular first molars typically present with 2 well-defined roots, a mesial root with two canals and a distal root with a wide oval canal or 2 round canals [3]. Apart from these presentations, wide variations of root and canal configuration of the mandibular first molars have been reported in the literature [5–13].

The mandibular first molars erupt at the age of 6-7 years and apical closure is usually completed by 8-9 years. The completion of canal differentiation commences at about 3–6 years after root completion [15]. Any disturbances in this differentiation could have resulted in this type of canal anatomy.

Systematic review of literature on canal morphology of the mandibular first molar by Valencia De Pablo et al. [18] and Ballulaya et al. [19] has not documented this rare morphology. But this morphological variation has been documented only once earlier in an *in vitro* study done by Reuben et al. [20]. Out of 125 samples of mandibular first molars from an Indian population, only one sample had a single root and single canal.  Table 1 illustrates variations in mandibular first molar according to different authors.

Radiographic examination is an essential component in endodontic treatment. The use of multiple preoperative radiographs or an additional radiographic view from a 20-degree mesial or distal projection increases the chances of detecting unusual root canal morphology [21].

Kottoor et al. [22, 23] and La et al. [24] have suggested the use of CBCT for the purpose of determining the root canal morphology in cases with aberrations. In this particular case, multiple radiographs in variable horizontal angulations clearly indicated the presence of single root and single canal.

The root canal was located at the centre of the pulpal floor and dentinal map was not evident. Searching for an extra canal in such cases could lead to excessive removal of dentin and even perforation. Since the single canal was large, the enlargement was done using a circumferential filing technique using ISO taper files.

## 4. Conclusion

This case report highlights the uncommon anatomy of mandibular first molar with a single root and single canal. Multiple preoperative radiographs, careful inspection of the tooth under dental operating microscopes, and the choice of cleaning and shaping technique suitable for this uncommon root canal anatomy enabled us to achieve success in this case.

## Figures and Tables

**Table 1:** The variations in mandibular first molar according to different authors.

Author/year	Materials and methods	Number of teeth	Variations reported	Distribution of canals	Number of roots
Martínez-Berná and Badanelli (1985) [6]	Case report	1	6 root canals	3 mesial, 3 distal	2 roots
Beatty and Interian (1985) [5]	Case report	1	5 root canals	2 mesial, 3 distal	2 roots
Bolger and Schindler (1988) [13]	Case report	1	“C” shaped canal		2 roots
Reeh (1998) [7]	Case report	1	7 root canals	4 mesial, 3 distal	2 roots
Baugh and Wallace (2004) [8]	Case report	1	Middle mesial canal	3 mesial, 2 distal	2 roots
Ghoddusi et al. (2007) [11]	Case report	1	Four canals in distal root	2 mesial, 4 distal	4 roots
Krithikadatta et al. (2010) [15]	Case report	1	2 canals	1 mesial, 1 distal	2 roots
Kottoor et al. (2011) [23]	Case report	1	Middle distal canal	2 mesial, 3 distal	2 roots
Attam et al. (2012) [12]	Case report	3	Radix entomolaris	Distolingual root in each case	3 roots
Subbiya et al. (2013) [10]	Case report	1	Four canals in mesial root	4 mesial, 2 distal	2 roots
Reuben et al. (2008) [20]	*In vitro *	1/125 (Indian population)	Single canal	Single canal (in the centre of the pulp chamber)	1 root

## References

[1] Ingle JI, Bakland LK, Baumgartner JC (2008). *Endodontics*.

[2] Cohen S, Hargreaves KM (2006). *Pathways of the Pulp*.

[3] Vertucci FJ (1984). Root canal anatomy of the human permanent teeth. *Oral Surgery Oral Medicine and Oral Pathology*.

[4] Skidmore AE, Bjorndal AM (1971). Root canal morphology of the human mandibular first molar. *Oral Surgery, Oral Medicine, Oral Pathology*.

[5] Beatty RG, Interian CM (1985). A mandibular first molar with five canals: report of case. *The Journal of the American Dental Association*.

[6] Martínez-Berná A, Badanelli P (1985). Mandibular first molars with six root canals. *Journal of Endodontics*.

[7] Reeh ES (1998). Seven canals in a lower first molar. *Journal of Endodontics*.

[8] Baugh D, Wallace J (2004). Middle mesial canal of the mandibular first molar: a case report and literature review. *Journal of Endodontics*.

[9] Kottoor J, Sudha R, Velmurugan N (2010). Middle distal canal of the mandibular first molar: a case report and literature review. *International Endodontic Journal*.

[10] Subbiya A, Kumar KS, Vivekanandhan P, Prakash V (2013). Management of mandibular first molar with four canals in mesial root. *Journal of Conservative Dentistry*.

[11] Ghoddusi J, Naghavi N, Zarei M, Rohani E (2007). Mandibular first molar with four distal canals. *Journal of Endodontics*.

[12] Attam K, Nawal RR, Utneja S, Talwar S (2012). Radix entomolaris in mandibular first molars in Indian population: a review and case reports. *Case Reports in Dentistry*.

[13] Bolger WL, Schindler WG (1988). A mandibular first molar with a C-shaped root configuration. *Journal of Endodontics*.

[14] Gopikrishna V, Bhargavi N, Kandaswamy D (2006). Endodontic management of a maxillary first molar with a single root and a single canal diagnosed with the aid of spiral CT: a case report. *Journal of Endodontics*.

[15] Krithikadatta J, Kottoor J, Karumaran CS, Rajan G (2010). Mandibular first molar having an unusual mesial root canal morphology with contradictory cone-beam computed tomography findings: a case report. *Journal of Endodontics*.

[16] Sabala CL, Benenati FW, Neas BR (1994). Bilateral root or root canal aberrations in a dental school patient population. *Journal of Endodontics*.

[17] Fava LRG, Weinfeld I, Fabri FP, Pais CR (2000). Four second molars with single roots and single canals in the same patient. *International Endodontic Journal*.

[18] De Pablo ÓV, Estevez R, Péix Sánchez M, Heilborn C, Cohenca N (2010). Root anatomy and canal configuration of the permanent mandibular first molar: a systematic review. *Journal of Endodontics*.

[19] Ballulaya SV, Vemuri S, Kumar PR (2013). Variable permanent mandibular first molar: review of literature. *Journal of Conservative Dentistry*.

[20] Reuben J, Velmurugan N, Kandaswamy D (2008). The evaluation of root canal morphology of the mandibular first molar in an Indian population using spiral computed tomography scan: an in vitro study. *Journal of Endodontics*.

[21] Fava LRG, Dummer PMH (1997). Periapical radiographic techniques during endodontic diagnosis and treatment. *International Endodontic Journal*.

[22] Kottoor J, Velmurugan N, Sudha R, Hemamalathi S (2010). Maxillary first molar with seven root canals diagnosed with cone-beam computed tomography scanning: a case report. *Journal of Endodontics*.

[23] Kottoor J, Velmurugan N, Surendran S (2011). Endodontic management of a maxillary first molar with eight root canal systems evaluated using cone-beam computed tomography scanning: a case report. *Journal of Endodontics*.

[24] La S-H, Jung D-H, Kim E-C, Min K-S (2010). Identification of independent middle mesial canal in mandibular first molar using cone-beam computed tomography imaging. *Journal of Endodontics*.

